# Stimulus data and experimental design for a self-paced reading study on emoji-word substitutions

**DOI:** 10.1016/j.dib.2022.108399

**Published:** 2022-06-18

**Authors:** Tatjana Scheffler, Lasse Brandt, Marie de la Fuente, Ivan Nenchev

**Affiliations:** a German Studies, Ruhr-Universität Bochum, Germany; b Department of Psychiatry and Psychotherapy, Charité Campus Mitte, Charité Universitätsmedizin Berlin, corporate member of Freie Universität Berlin, Humboldt- Universität zu Berlin, and Berlin Institute of Health, Berlin, Germany; c Department of Linguistics, University of Potsdam, Germany

**Keywords:** Emojis, Self-paced reading, Lexical ambiguity, Homonymy, Processing

## Abstract

This data paper presents the experimental design and stimuli from an online self-paced reading study on the processing of emojis substituting lexically ambiguous nouns. We recorded reading times for the target ambiguous nouns and for emojis depicting either the intended target referent or a contextually inappropriate homophonous noun. Furthermore, we recorded comprehension accuracy, demographics and a self-assessment of the participants’ emoji usage frequency. The data includes all stimuli used, the raw data, the full JavaScript code for the online experiment, as well as Python and R code for the data analysis. We believe that our dataset may give important insights related to the comprehension mechanisms involved in the cognitive processing of emojis. For interpretation and discussion of the experiment, please see the original article entitled “The processing of emoji-word substitutions: A self-paced-reading study”.

## Specification Table

**Table:** 

Subject area	Experimental and Cognitive Psychology
More specific subject areas	Psycholinguistics, visual communication, experimental linguistics, emojis
Type of data	Items used in the study, raw results, JavaScript code for the online experiment, Python and R code used in the data analysis
How data was acquired	Online browser-based experiment using the open-source platform _magpie
Data format	CSV, iPython notebook, R, JavaScript, HTML
Description of data collection	The reading times for target items in three experimental conditions (word string, matching emoji, homophone emoji) from 63 monolingual German-speaking participants were recorded; comprehension questions measured if the intended meaning has been retrieved; participants self-accessed emojis usage frequency.
Data source location	Ruhr-Universität Bochum, Bochum, Germany
Data accessibility	Data is in the online repository, https://osf.io/d34y5/, doi:10.17605/OSF.IO/D34Y5
Related research article	Tatjana Scheffler, Lasse Brandt, Marie de la Fuente, and Ivan Nenchev. The processing of emoji-word substitutions: A self-paced-reading study. Computers in Human Behavior 127. 2022. doi: https://doi.org/10.1016/j.chb.2021.107076

## Value of the Data

• The following data gives insight into the cognitive processing of emojis substituting nouns;
• The dataset can be used in new research – in terms of methodology, it can be expanded to include new experimental conditions in order to explore the processing of other types of emojis and other positions of emojis within the test sentences. Furthermore, the study can be conducted with new respondent groups in oder to explore demographic differences / national differences / international differences etc., of emoji processing. The current dataset can also be used to develop an eye-tracking study or an fMRI study.
• This data can be used as a historical reference point for the processing of emoji-word substitutions in 2020.

## 1 Data

The data presented in this article is associated with the published research paper entitled “The processing of emoji-word substitutions: A self-paced-reading study” [1]. The data set includes 9 files: experimental-items.pdf, emoji-homophones-data.csv, emoji-homophones-data-translated.csv, experiment-code.zip, experiment-instructions.pdf, emoji-homophones-analysis.ipynb, emoji-homophones-lmm.ipynb, homophones-lmm.nb.html and homophones-lmm.Rmd.

The experimental-items.pdf file contains both the experimental stimuli items and the filler items in German as used in the original experiment, as well as their English translations. Each test item is presented in a table form with three columns: name, sentence/translation, emoji. The sentence/translation column contains a short introductory scene setting sentence and a sentence containing the target lexically ambiguous noun, which is underlined. In the original experiment, this target noun was sometimes replaced with a corresponding emoji or an emoji depicting the contextually inappropriate homophone noun (based on the experimental condition). Both emojis are presented in the emoji column. The 26 filler items used the same item structure but different (sometimes matching, sometimes ill-fitting) emojis.

The emoji-homophones-data.csv file contains the raw data collected during the online experiment in a table form. The columns "sona" and "comments" have been deleted from the original 29 columns, as they could potentially partially identify the participants.

The column “experiment_id” contains a constant number identifying the experiment in the database (“2”), while the columns “submission_id” and “participant_id” list sequential resp. randomly generated numerical ids for each participant in the experiment. In order to ensure that participants saw each item only once, 3 groups (A, B, C) were created, to which the participants were randomly assigned (column “participant_group”). Since trial orders were also randomized within participants, the column “trial_number” represents the exact order in which the items were presented to the participant. The column “trial_name” allows a distinction between training items (called “spr_tryout”) and test items.

The columns “itemname”, “sentence”, “question”, “option1”, “option2”, “correct_answer” and “QUD” identify the presented single test item.

Reading times for each single token (word or emoji) are recorded in the column “reaction_times”, separated by the “|” character (the tokenization as presented is also reflected in the experimental “sentence”). Column “time_spent” lists the cumulative reading time for the whole item. The column “response” contains participants´ answers to the comprehension question.

The columns “startDate”, “startTime”, “endTime” and “timeSpent” all contain cumulative information on the reaction times for the whole experiment.

The columns “underline” and “wordPos” are identical for all participants and indicate the presentation mode during the experiment: the target word was not underlined (“none”) and each word token was shown in the “same” position on the screen.

The columns “age”, “gender”, “education” and “languages” contain the demographic characteristics of the participants obtained in a post-test survey. Furthermore, the column “emoji” contains participants’ self-assessment of their emoji usage frequency on a five-point scale (*nie* “never”, *selten* “rarely”, *manchmal* “sometimes”, *häufig* “often”, *beinahe immer* “almost always”). Translations for all stimuli contained in the file “emoji-homophones-data.csv” are provided in “experimental-items.pdf”. The file “emoji-homophones-data-translated.csv” provides English translations for all other codes in the raw data file.

The experiment-code.zip file contains the JavaScript code used for the online experiment. We included translations of the instructions in English.

The files emoji-homophones-analysis.ipynb, emoji-homophones-lmm.ipynb, homophones-lmm.nb.html and homophones-lmm.Rmd contain the Python and R code which we used to analyze the raw data.

## 2 Experimental Design, Materials and Methods

### 2.1 Participants

Here we present the complete dataset from 63 monolingual German-speaking participants (51 female, 12 male; aged 18-75, Ø=25.0) who participated in the study. They were recruited via the University of Potsdam's cognitive science subject pool^1^ and word of mouth. The participants who were undergraduate students at the University of Potsdam received study credit for participation. Participants reported normal or corrected vision and no language-related impairments.

### 2.2 Materials

We carefully selected 15 common German lexically ambiguous words, where both meanings represent concrete objects and are therefore representable as emojis. Using the two possible meanings of the target nouns, we constructed 15 paired scenarios resulting in a total of 30 experimental contexts. In each context, we either presented the target noun as a word string or replaced it with either an appropriate emoji or an emoji depicting the homophone meaning, for a total of 90 different experimental item variants.

The emojis were selected from the the freely licensed Twitter emoji icon set Twemoji, and we showed them to participants as embedded images in order to ensure consistency across devices and operating systems. We first carried out a separate online questionnaire to find the most appropriate emojis for each ambiguous target noun. 45 participants (completely distinct from the participants in the actual experiment) completed this pretest. We showed them simple sentences with a capitalized word as in (1) and asked participants to select the most appropriate emoji out of three presented options. This pretest ensured that readers are able to associate the emoji with the target noun. Where several different emojis exist for one object (such as (rodent) mouse:  or ), we used the most frequently chosen emoji from the pretest questionnaire to construct the experimental items.

(1) Die MAUS ist ein Nagetier.

‘The MOUSE is a rodent’

Each experimental item consisted of an introductory scene-setting sentence, a target sentence containing the critical ambiguous noun and a comprehension question. The role of the introductory scene-setting sentence was to disambiguate the critical noun. Care was taken in order for the experimental sentences to have an equal syntactic structure. Each sentence started with a name, verb, and two adverbs. This was always followed by a determiner and the target noun, and finally a spillover region of three words. Note that the target noun was always placed sentence-medially. Not placing the target noun at the end of the sentence was motivated by previous research which indicates that emojis placed in the sentence final position have longer reading times [2]. This also allows for late-manifesting comprehension problems to appear in the spillover region.

Since the critical noun is semantically ambiguous, it was presented in one of three conditions: as a word string, as an emoji which depicts the critical noun's contextually appropriate meaning, or as an emoji which depicts a homophonous meaning to the intended noun. In order to check whether the participants were able to grasp the intended meaning of the experimental items, we presented a follow-up yes-no-question. Half of the comprehension questions were set up to be answered positively and the other half were set up to be answered negatively. In Table 1 (based on [1], Table 1) we present an example of the target noun “mouse” in its two experimental contexts. The two contexts are introduced by the different scene-setting sentences. While in context (a),  is the matching emoji, it is the homophone in context (b).

**Table 1:** Paired experimental items for (rodent vs. computer) ‘mouse’ (word/WO, matching emoji/MA, or homophone emoji/HO).

In addition to the experimental items, we also constructed 26 filler items which were syntactically more diverse than the experimental items. 19 of these items contained either appropriate or inappropriate emojis in a sentence medial position. The remaining 5 filler items contained only words.

In order to ensure that the participants remain blind to the homophone phenomenon during the experiment, we split the participants into three groups (A, B and C). In each group each experimental context was only seen in one condition. Furthermore, we made sure that each participant saw each emoji at most once. That means that for each pair of contexts with homophonous target nouns, a participant either saw one non-matching homophone emoji and one word string, or two different emojis that each matched their context. For example, for the items shown in Table 1, participant group A saw  in context (a) and the word “mouse“ in context (b); group B saw “mouse“ in context (a) and  in context (b); and group C saw  in context (a) and  in context (b). In total, we recorded N=1890 observations, not including fillers. The fillers were the same for all participant groups.

### 2.3 Procedure

The experiment was implemented using the open source platform _magpie for browser-based psycholinguistic experiments^2^ and deployed over the internet. After opening the experiment, the subject gave consent to participation. They were instructed to read the presented sentences at a normal reading speed and answer the comprehension questions after each item. The sentences were presented on the screen word by word, each word is advanced by pressing the space bar and these key presses are time logged. In order to familiarize and habituate the participants with the self-paced reading task, the experiment started with two training items exhibiting the same structure as the experimental items and containing matching emojis instead of a noun. After finishing the training items, the actual experiment began and each participant read a total of 56 items which consisted of 30 experimental items and 26 filler items. Using a final post-test survey, we collected general demographic information on age, gender, language and education level. Furthermore, the participants self-assessed their emoji usage frequency on a five-point rating scale.

## Ethics Statements

The participants gave informed consent prior to their participation in the online experiment, and agreed to the storage, processing, and sharing of their data for scientific purposes. According to the standards of the German Research Foundation (DFG), no approval from an ethics committee or institutional review board is needed for acquiring behavioral linguistic data from healthy adult participants.

## CRediT Author Statement

**Tatjana Scheffler:** Conceptualization, Methodology, Software, Formal Analysis, Writing – original draft; **Lasse Brandt:** Methodology; **Marie de la Fuente:** Methodology, Data Curation, Investigation; **Ivan Nenchev:** Conceptualization, Methodology, Writing – original draft.

## Declaration of Competing interest

The authors declare that they have no known competing financial interests or personal relationships that could have appeared to influence the work reported in this paper.

## Data Availability

Emoji Homophones (Original data) (OSF).

## Footnotes

1 https://uni-potsdam.sona-systems.com

2 https://magpie-ea.github.io/magpie-site/
